# Genome-wide identification, characterization and expression analysis of the HD-Zip gene family in the stem development of the woody plant *Prunus mume*

**DOI:** 10.7717/peerj.7499

**Published:** 2019-08-08

**Authors:** Lulu Li, Tangchun Zheng, Xiaokang Zhuo, Suzhen Li, Like Qiu, Jia Wang, Tangren Cheng, Qixiang Zhang

**Affiliations:** 1 Beijing Key Laboratory of Ornamental Plants Germplasm Innovation & Molecular Breeding, Beijing Forestry University, Beijing, China; 2 National Engineering Research Center for Floriculture, Beijing Forestry University, Beijing, China; 3 Beijing Laboratory of Urban and Rural Ecological Environment, Beijing Forestry University, Beijing, China; 4 Engineering Research Center of Landscape Environment of Ministry of Education, Beijing Forestry University, Beijing, China; 5 Key Laboratory of Genetics and Breeding in Forest Trees and Ornamental Plants of Ministry of Education, Beijing Forestry University, Beijing, China; 6 Beijing Advanced Innovation Center for Tree Breeding by Molecular Design, Beijing Forestry University, Beijing, China

**Keywords:** *Prunus mume*, Genetic evolution, Expression pattern, Stem development, HD-Zip TFs

## Abstract

The homeodomain-leucine zipper (HD-Zip) gene family, a group of plant-specific transcriptional factors (TFs), participates in regulating growth, development, and environmental responses. However, the characteristics and biological functions of HD-Zip genes in *Prunus mume*, which blooms in late winter or early spring, have not been reported. In this study, 32 HD-Zip genes, named *PmHB1*–*PmHB32* based on their chromosomal positions, were identified in the genome of *P. mume*. These genes are distributed among seven chromosomes and are phylogenetically clustered into four major groups. Gene structure and motif composition were mostly conserved in each group. The Ka/Ks ratios showed that purifying selection has played a leading role in the long-term evolution of the genes, which maintained the function of this family. MicroRNA target site prediction indicated that the genes of the HD-Zip III subfamily may be regulated by miR165/166. Expression pattern analysis showed that the 32 genes were differentially expressed across five different tissues (leaf, flower bud, stem, fruit, and root) and at different stages of stem and leaf-bud development, suggesting that 10 of the genes may play important roles in stem development. Protein–protein interaction predictions showed that the subfamily III genes may regulate vascular development and shoot apical meristem (SAM) maintenance. Promoter analysis showed that the HD-Zip III genes might be involved in responses to light, hormones, and abiotic stressors and stem development. Taken together, our results provide an overview of the HD-Zip family in *P. mume* and lay the foundation for the molecular breeding of woody ornamental plants.

## Introduction

During growth and development, plants are controlled by many transcriptional factors. The plant-specific homeodomain-leucine (HD-Zip) proteins transcriptional factors affect various biological processes including development, cell division, and responses to abiotic stresses in plants (Elhiti & Stasolla, 2014). HD-Zip proteins are characterized by a C-terminal homeodomain (HD) domain, which is responsible for sequence-specific DNA-binding, and an adjacent leucine zipper (LZ), which facilitates protein–protein interactions. Based on sequence similarity and additional conserved motifs, HD-Zip proteins can be classified into four subfamilies: HD-Zip I, II, III, and IV (Ariel et al., 2007). Compared with HD-Zip I proteins, HD-Zip II proteins contain conserved CPSCE motifs and N-terminal consensus sequences. Both HD-Zip III and HD-Zip IV proteins have a steroidogenic acute regulatory protein (StAR)-related lipid transfer domain (START), which has an unknown function in plants. Compared with HD-Zip III proteins, HD-Zip IV proteins lack the C-terminal MEKHLA motif, but contain the PAS domain, which may be involved in the circadian clock and signal transduction (Mukherjee & Bürglin, 2006; Hefti et al., 2004). To date, many HD-Zip genes have been identified, and many attempts have been made to elucidate the functions of various HD-Zip genes in several species, such as *Arabidopsis thaliana* (Henriksson, 2005; Nakamura et al., 2006; Ciarbelli et al., 2008), rice (*Oryza sativus*) (Agalou et al., 2008), maize (*Zea mays*) (Javelle et al., 2011), tomato (*Solanum lycopersicum*) (Zhang et al., 2014a; Zhang et al., 2014b), *Brassica rapa* (Khan et al., 2018), cotton (*Gossypium hirsutum*) (He et al., 2018), soybean (*Glycine max*) (Chen et al., 2014), foxtail millet (*Setaria italica*) (Chai et al., 2018), sunflower (*Helianthus annuus*) (Manavella et al., 2010; Dezar et al., 2011), poplar (*Populus trichocarpa*) (Hu et al., 2012), grape (*Vitis vinifera*) (Jiang et al., 2014), pear (*Pyrus betulifolia*) (Wang et al., 2015) and peach (*Prunus persica*) (Zhang et al., 2014a; Zhang et al., 2014b).

Genes within the same subfamily tend to exhibit functional conservation due to structural similarities. Several members of the HD-Zip I subfamily are involved in responses to abiotic stresses and phytohormones, embryogenesis, and de-etiolation and response to blue light. These genes include *AtHB6*, *AtHB7*, and *AtHB12* from *Arabidopsis* (Söderman, Mattsson & Engström, 1996; Söderman et al., 1999; Lee et al., 2001), which are induced by drought and abscisic acid (ABA), and *HaHB4* from sunflower, which responds to methyl jasmonate (MeJA), ethylene, and drought stress (Manavella et al., 2010). Furthermore, overexpression of *AtHB13* in *Arabidopsis* and *HaHB1* in sunflower can significantly improve the cold resistance of transgenic plants (Cabello, Arce & Chan, 2012); the maize HD-Zip gene *Zmhdz10* enhances the drought resistance of transgenic rice and *Arabidopsis* (Zhao et al., 2014). In contrast, *MtHB1* reduces the drought resistance of alfalfa by inhibiting lateral root formation (Tina et al., 2017). Moreover, *ATHB52* can respond to light and regulate photomorphogenesis and de-etiolation (Henriksson, 2005), and *AtHB1* responds to short-day photoperiods and promotes hypocotyl and root elongation (Capella et al., 2015). In addition to the above functions, HD-Zip I genes are associated with the development of plant organs. For example, *Arabidopsis hb21/hb40/hb53* triple mutants produced more lateral buds than wild-type plants under short-day conditions, which showed that *AtHB21/AtHB40/AtHB53* negatively regulate bud formation (Gonzalez-Grandio et al., 2017).

HD-Zip II genes have key roles in plant responses to light and auxin signalling. For instance, *ATHB4* and *HAT3* from *Arabidopsis*, two auxin-induced genes, are involved in shade-induced growth (Bou-Torrent et al., 2012). The sunflower HD-Zip II gene *HaHB10* responded to dark and light conditions and was associated with the induction of flowering (Dezar et al., 2011). In addition, there is evidence showing that many HD-Zip II genes are relevant to the regulation of organ growth and development. The *AtHAT3*, *AtHB2*, and *AtHB4* genes co-regulate shoot apical meristem (SAM) and cotyledon development in *Arabidopsis* seedlings (Turchi et al., 2013). The *Arabidopsis* loss-of-function mutant *athb4/hat3* results in severely abaxialized leaves (Bou-Torrent et al., 2012).

HD-Zip class III proteins act as regulators of apical meristem development, embryogenesis, lateral organ initiation, and vascular bundle development. The functions of some HD-Zip III genes have been investigated both in herbaceous and woody plants. There are five (*REV*, *PHB*, *PHV*, *CAN/ATHB-15*, and *ATHB-8*) and eight (*PtrHB1–8*) HD-Zip III genes in *Arabidopsis* and *Populus*, respectively (Prigge et al., 2005; Hu et al., 2012). *REV*, *PHB* and *PHV*, along with *KANADI*, are believed to control the abaxial-adaxial patterning of lateral organs, such as leaves and embryos (Emery et al., 2003a). All five members of the HD-Zip III subfamily are thought to be directly involved in vascular development in *Arabidopsis* by up- or down-regulation. For example, *REV*, *PHB*, and *PHV* promote, while *CAN* and *ATHB-8* inhibit, the formation of interfascicular cambium at the base of the inflorescence stem (Baima, 2001; Prigge et al., 2005). *PopREVOLUTA* (*PRE, PtrHB2*), a *REV*- homologous gene in poplar, plays a key role in regulating the initiation of cambia and the pattern of secondary vascular tissues (Robischon et al., 2011). In contrast, *PtrHB5* (*PCN*), a *CAN*-homologous gene, inhibits the development of secondary vascular tissue (Du et al., 2011). A *PtrHB4* mutation caused a defect in interfascicular cambium in transgenic poplar (Zhu et al., 2018). Because of changes in vascular patterns, some transgenic plants exhibit various morphological characteristics, such as tortuous stems and leaves, dwarfism, and shortened internodes (Du et al., 2011; Zhu et al., 2013; Zhu et al., 2018).

HD-Zip class IV proteins control epidermal cell differentiation and cuticle and trichome formation. Most HD-Zip IV genes are specifically expressed in epidermal or subepidermal layers: these genes include *Arabidopsis GL2* (*GLABRA2*), *ATHB-10*, *ANL2* (*ANTHOCYANINLESS 2*), *ATML1* (*ARABIDOPSIS THALIANA MERISTEM LAYER 1*), and *PDF2* (*PROTODERMALFACTOR 2*) (Abe, 2003) and *OCL1* (*OUTER CELL LAYER 1*) and *OCL4* (*OUTER CELL LAYER 4*) from maize (Vernoud et al., 2009; Depègefargeix et al., 2011). The *atml1/pdf2* double mutant exhibits a few defects in shoot epidermal cell differentiation in *Arabidopsis*. *GL2* and *ATHB-10* suppress hair formation to determine trichome and root-hair patterning in *A. thaliana* by coordinating with single-repeat R3-MYBs (Ohashi et al., 2010). The latest findings suggest that *AaHD8* not only promotes the initiation of glandular trichomes but also facilitates leaf cuticle development in *Artemisia annua* (Yan et al., 2018).

*Prunus mume* is an economically important fruit and ornamental plant that has been cultivated in China for thousands of years; it is widely distributed and used in East Asia. *P. mume* has acquired favourable ornamental characteristics, including colourful corollas, a pleasant fragrance, and various flower shapes. In addition, it has different types of plant architecture including the upright, weeping, and tortuous types (Chen, 1996). It is important to improve plant architectural traits that are associated with the development of tissues and organs. HD-Zip transcriptional factors can not only enhance resistance to abiotic stress but also improve morphological features to control the growth and development of multiple tissues and organs, such as the apical meristem, vascular tissue, epidermal tissue, and lateral organs. Compared to abundant annotations of *Arabidopsis* HD-Zip genes, no *P. mume* HD-Zip has been analysed in detail. In this study, we identified 32 HD-Zips genes from *P. mume* and performed detailed structural, evolutionary, miRNA165/166 target gene, and expression pattern analyses in different organs and at different stem development stages. Our study provides an overview of the HD-Zip family in *P. mume* and information to aid understanding of HD-Zip functions in woody plants.

## Materials & Methods

### HD-Zip gene identification in *P. mume* and other species

Candidate genes of the HD-Zip family were extracted from the *A. thaliana*, *P. mume* (Zhang et al., 2012), *Prunus avium* (Shirasawa et al., 2017), and *Prunus persica (International Peach Genome et al., 2013)* genome databases using the software HMMER (Johnson, Eddy & Portugaly, 2010) based on a Hidden Markov Model (HMM) of the HD-domain profile (PF00046). The genome database of *P. mume* was downloaded from NCBI (https://www.ncbi.nlm.nih.gov/genome/13911) (Zhang et al., 2012). The genomes of *P. avium* and *P. persica* were downloaded from the GDR database (https://www.rosaceae.org/), and the genome of *A. thaliana* was downloaded from NCBI databases (https://www.ncbi.nlm.nih.gov/). All obtained protein sequences were further analysed by SMART (http://smart.embl-heidelberg.de/) and CD-search (http://www.ncbi.nlm.nih.gov/Structure/cdd/wrpsb.cgi) softwares to confirm the presence of the leucine-zipper (LZ) domains. Sequences lacking the LZ domains were discarded.

### Gene classification and phylogenetic analysis

Multiple sequence alignments of the full-length HD-Zip protein sequences from *P. mume*, *A. thaliana, P. avium,* and *P. persica* were performed by the NAFFT 7 program (Katoh, Rozewicki & Yamada, 2017). The maximum-likelihood (ML) phylogenetic tree was constructed using MEGA 6.0 under the Jones–Taylor–Thornton (JTT) amino acid substitution model with 1,000 bootstrap replicates. The full-length sequences of all HD-Zip proteins used in this study is provided in Data S1.

### Gene characteristics, structure, and conserved motif analyses

The genomic sequences, ID numbers, and chromosomal locations of the *P. mume* HD-Zip genes were obtained from the *P. mume* genome. The physical parameters of putative proteins, including the lengths of the amino-acid sequences (aa), molecular weights (MWs), theoretical isoelectric points (pIs), and grand averages of hydropathicity (GRAVYs), were calculated using the online ExPASy tool (http://www.expasy.org/tools/protparam.html). The gene structure display server (GSDS) program (http://gsds.cbi.pku.edu.cn/) was applied to illustrate the exon–intron structures by alignment of the CDS with the DNA sequences of individual HD-Zip genes. The orthologues of *P. mume* HD-Zip in *Arabidopsis* mapping and GO annotation were conducted using the software Blast2GO (Conesa et al., 2005). The detailed information of all orthologues is provided in Data S2.

The online MEME program was used to display the motif structures of HD-Zip proteins (http://meme-suite.org/tools/meme) using the default parameters, except that the optimum motif widths were set from 6 to 200 residues. SMART (http://smart.embl-heidelberg.de) and CD-search (http://www.ncbi.nlm.nih.gov/Structure/cdd/wrpsb.cgi) web servers were used for structural motif annotation.

### Chromosomal location and gene duplication analyses

The chromosomal location information of HD-Zip genes was obtained from the *P. mume* genome database, and the chromosomal location map was produced by TBtools (Chen et al., 2018). Tandem duplicated genes were identified, and paralogues were genes separated by five or fewer genes in a 100-kb region, as proposed by Tu et al. (2010). Identification of segmentally duplicated genes was conducted using the Plant Genome Duplication Database (http://chibba.agtec.uga.edu/duplication/index/downloads). Searches for transposons and retrotransposons in DNA sequences 10-kb upstream and downstream of each HD-Zip gene were conducted with RepeatMasker software (http://www.repeatmasker.org/cgi-bin/WEBRepeatMasker/).

### Estimation of Ka/Ks ratios

The non-synonymous substitution (Ka) and synonymous substitution (Ks) values of duplicated pairs were calculated on PAL2NAL (http://www.bork.embl.de/pal2nal) (Suyama, Torrents & Bork, 2006; Yang, 2007) after the alignment of protein sequences. The duplication time of duplicated genes was calculated by the following formula: *T* = *Ks*∕2*λ* × 10^6^ Mya (*λ* = 1.5 × 10^−8^ for dicots) (Blanc & Wolfe, 2004).

### Cis-elements in promoters and miR165/166 target site prediction

The online database PlantCARE (http://bioinformatics.psb.ugent.be/webtools/plantcare/html/) (Lescot, 2002) was employed to investigate putative cis-regulatory elements in the sequences approximately 1,500-bp upstream of the translational start site (ATG).

Full-length HD-Zip nucleic acid sequences (including introns and UTRs) were obtained, and psRNATarget software (http://plantgrn.noble.org/psRNATarget/?function) was used to predict miR165 and miR166 target sites (Wang et al., 2014b).

### Expression profile analysis based on transcriptome data

To gain insight into the tissue-specific expression patterns of *PmHB* genes, our previous RNA-Seq data (GEO No. GSE40162) were used to generate a heat map based on the reads per kilobase per million (RPKM) values of individual gene in all tissue samples. HD-Zip gene expression levels were analysed in five organs of *P. mume*: roots, stems, leaves, flower buds, and fruits (Zhang et al., 2012). HD-Zip gene expression quantities in flower buds at the endodormancy stage (ED) and natural flush stage (NF) were obtained by RNA sequencing during bud dormancy (Zhang et al., 2018). The heat maps were illustrated using the HemI 1.0 software with the default settings (http://hemi.biocucukoo.org/faq.php).

### Preparation of plant materials

*P. mume* cv. ‘Fei Lve’ was cultivated in the greenhouse of Beijing Forestry University. To investigate the expression of *PmHB*s, leaf buds and young stems at different developmental stages were sampled from one-year-old branches (Fig. S1). All samples were collected and frozen immediately in liquid nitrogen and then stored at −80 °C until needed for RNA extraction.

### RNA extraction and qRT-PCR

Total RNA was extracted from leaf buds and stem tissues using the RNA extraction kit (Tiangen, Beijing, China). After testing for concentration and quality, 500 ng total RNA was used to synthesize first-strand cDNA using the PrimeScript RT Reagent kit (TaKaRa, Dalian, China). The SYBR Premix *Ex Taq* II kit (TaKaRa, Dalian, China) was used for qRT-PCR according to the manufacturer’s instructions. *PP2A* and *actin* were selected as reference genes based on previous reports (Wang et al., 2014a; Xu et al., 2015). Three biological replicates were carried out, and expression levels were calculated by the 2^−ΔΔ*Ct*^ method (Schmittgen & Livak, 2008). Gene-specific primers used in the qRT-PCR analyses were designed by IDT (https://sg.idtdna.com/scitools/Applications/RealTimePCR/) and are listed in Table S1.

**Table 1:** Features of 32 HD-Zip genes in *P. mume* and their sequence characteristic.

**Gene name**	**Gene ID**	**Locus**	**Start**	**End**	***pI***	**MW**	**Strand**	**Subcellular localization**	**Gene length (bp)**	**ORF (bp)**	**Protein length (aa)**	**Exons**	**Introns**	**Homolog in *Arabidopsis***
*PmHB1*	*Pm001037*	Pm1	6524197	6530678	6.02	92.54	–	Nucleus	6482	2527	842	18	17	*REV*
*PmHB2*	*Pm002097*	Pm1	16624414	16625975	5.42	38.08	–	Nucleus	1562	1012	337	3	2	*ATHB-6*
*PmHB3*	*Pm002327*	Pm1	18696993	18698133	8.62	31.60	–	Nucleus	1141	856	285	3	2	*HAT22*
*PmHB4*	*Pm004476*	Pm2	5264456	5265317	5.46	28.82	–	Nucleus	862	763	254	2	1	*ATHB-6*
*PmHB5*	*Pm005163*	Pm2	9571242	9576314	5.83	92.89	–	Nucleus	5073	2521	840	18	17	*ATHB-8*
*PmHB6*	*Pm005455*	Pm2	11136937	11138132	6.24	35.78	–	Nucleus	1196	949	316	3	2	*HAT22*
*PmHB7*	*Pm006530*	Pm2	17359670	17361143	5.72	32.89	–	Nucleus	1474	871	290	3	2	*ATHB-13*
*PmHB8*	*Pm006578*	Pm2	17692588	17694392	4.69	36.36	–	Nucleus	1805	964	321	3	2	*HAT5*
*PmHB9*	*Pm008838*	Pm2	36540330	36541338	9.31	27.57	–	Nucleus	1009	724	241	3	2	*HAT22*
*PmHB10*	*Pm009758*	Pm3	782406	785901	5.96	82.14	–	Nucleus	3496	2251	750	10	9	*ATML1*
*PmHB11*	*Pm010515*	Pm3	4909848	4916241	5.76	86.72	–	Nucleus	6394	2374	791	18	17	*ATHB-14*
*PmHB12*	*Pm011328*	Pm3	10393933	10394448	7.1	19.97	–	Nucleus	516	514	171	1	0	*ATHB-52*
*PmHB13*	*Pm013001*	Pm4	1041360	1045089	6.3	72.62	–	Nucleus	3730	1957	652	10	9	*HDG11*
*PmHB14*	*Pm013438*	Pm4	4125685	4131654	6.06	92.11	–	Nucleus	5970	2512	837	18	17	*ATHB-15*
*PmHB15*	*Pm013549*	Pm4	4819146	4823243	5.99	84.58	–	Nucleus	4098	2272	757	11	10	*GLABRA 2*
*PmHB16*	*Pm015192*	Pm4	17990442	17995263	5.78	78.05	–	Nucleus	4822	2137	712	12	11	*HDG2*
*PmHB17*	*Pm015729*	Pm4	21131194	21133120	4.95	30.69	–	Nucleus	1927	820	273	3	2	*HAT5*
*PmHB18*	*Pm015861*	Pm4	21777276	21778882	6.57	35.05	–	Nucleus	1607	925	308	3	2	*HAT7*
*PmHB19*	*Pm017678*	Pm5	13200909	13202636	6.96	33.31	–	Nucleus	1728	898	299	4	3	*HAT3*
*PmHB20*	*Pm017987*	Pm5	15486990	15492069	6	90.37	–	Nucleus	5080	2488	829	9	8	*ANL2*
*PmHB21*	*Pm018314*	Pm5	17547953	17548796	4.93	27.76	–	Nucleus	844	724	241	2	1	*ATHB-7*
*PmHB22*	*Pm019365*	Pm5	23522721	23527271	6.03	88.19	–	Nucleus	4551	2425	808	9	8	*ANL2*
*PmHB23*	*Pm019693*	Pm5	25211414	25212172	7.82	25.37	–	Nucleus	759	658	219	2	1	*ATHB-12*
*PmHB24*	*Pm023161*	Pm7	2755202	2759847	5.42	92.33	–	Nucleus	4646	2494	831	11	10	*HDG5*
*PmHB25*	*Pm023405*	Pm7	4736073	4737543	7.62	34.88	–	Nucleus	1471	934	311	4	3	*HAT4*
*PmHB26*	*Pm024816*	Pm7	14444420	14446077	4.64	36.77	–	Nucleus	1658	988	329	3	2	*HAT5*
*PmHB27*	*Pm025254*	Pm7	16763051	16766231	6.3	78.27	–	Nucleus	3181	2128	709	10	9	*HDG11*
*PmHB28*	*Pm025933*	Pm8	6532622	6534440	8.86	38.76	–	Nucleus	1819	1054	351	4	3	*HOX11*
*PmHB29*	*Pm026270*	Pm8	8869226	8870539	8.31	26.16	–	Nucleus	1314	676	225	3	2	*ATHB-51*
*PmHB30*	*Pm026578*	Pm8	10635982	10637531	6.47	24.76	–	Nucleus	1550	643	214	3	2	*ATHB-40*
*PmHB31*	*Pm029201*	scaffold205	1964174	1967025	9.13	25.62	–	Nucleus	2852	685	228	3	2	*HOX3*
*PmHB32*	*Pm030054*	scaffold427	2076	3624	6.32	40.29	–	Nucleus	1549	1102	367	4	3	*HOX11*

## Results

### Identification, characteristics, and phylogenetic analysis in *P. mume* and other species

**Figure 1:**
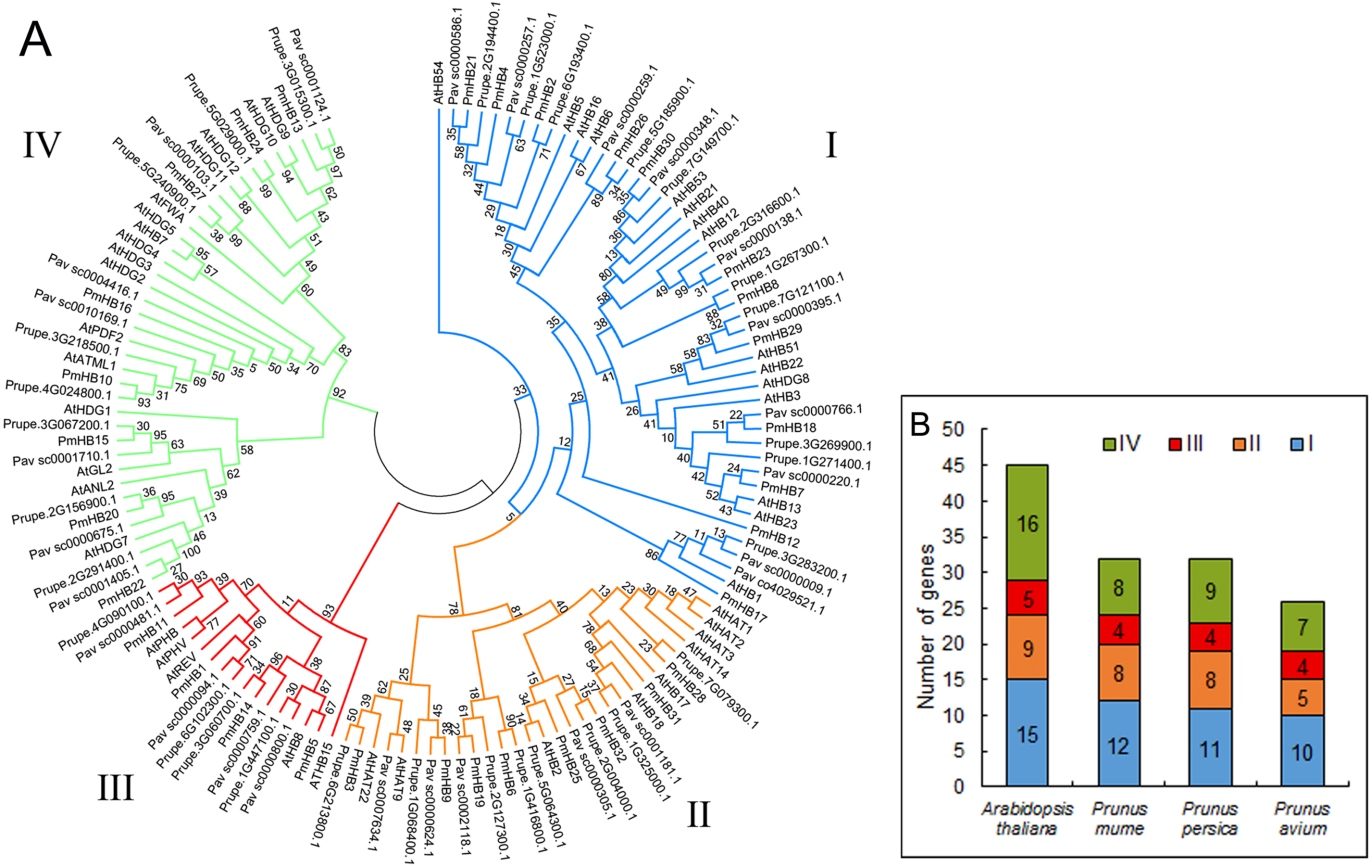
Phylogenetic tree analysis of HD-Zip sequences from *P. mume* and other plant species. (A) Phylogenetic tree of HD-Zip proteins in *P. mume*; the Group I, Group II, Group III, and Group IV subfamilies are indicated by blue, orange, red, and green branch lines, respectively. At, *A. thaliana*; Pm, *P. mume*; Pav, *P. avium*; Prupe, *P. persica*; The phylogenetic tree was produced with MEGA 6.0 by the maximum likelihood (ML) method under the Jones-Taylor-Thornton (JTT) amino acid substitution model with 1,000 bootstrap replications. (B) The gene numbers of subfamily in *A. thaliana, P. mume, P. avium,* and *P. persica*.

In total, 32, 32, 26, and 45 putative proteins containing the HD and LZ domains were identified in *P. mume*, *P. persica*, *P. avium*, and *A. thaliana*, respectively. The putative proteins of *P. mume* were named *PmHB1*–*PmHB32* based on their chromosomal positions in the order of successive chromosomes (Table 1). Among the putative PmHBs, physicochemical characteristics were variable. Their encoded proteins had diverse lengths, from 171 aa to 842 aa, with an average length of 463 aa. The molecular weights ranged between 11.98 kDa and 92.32 kDa, and the isoelectric points ranged from 4.64 to 9.31. The number of HD-Zip genes in *P. mume* was 0.7-fold less than that in *A. thaliana* (45), whereas it was similar to that in *P. persica* (32) and less than that in *P. avium* (26). To investigate the classification of HD-Zip proteins in *P. mume* and their evolutionary relationships in *P. mume*, *P. persica*, *P. avium*, and *A. thaliana*, full-length HD-Zip proteins were used to construct a maximum-likelihood phylogenetic tree using MEGA software (Fig. 1A). The HD-Zip proteins of the four species were divided into four subfamilies (HD-Zip I to IV), and the proportions of each subfamily were similar in all four species (Fig. 1B). The HD-Zip I gene family was the largest subfamily in *P. mume* (37.5%), *P. persica* (34.4%), and *P. avium* (38.5%), while HD-Zip IV genes composed the largest proportion in *A. thaliana* (35.6%). HD-Zip III genes had the fewest representatives in all four species: there were 4 (12.5%), 4 (12.5%), 4 (15.4%), and 5 (11.1%) members in *P. mume*, *P. persica*, *P. avium*, and *A. thaliana*, respectively.

### Chromosomal location and gene duplication of *PmHBs*

**Figure 2:**
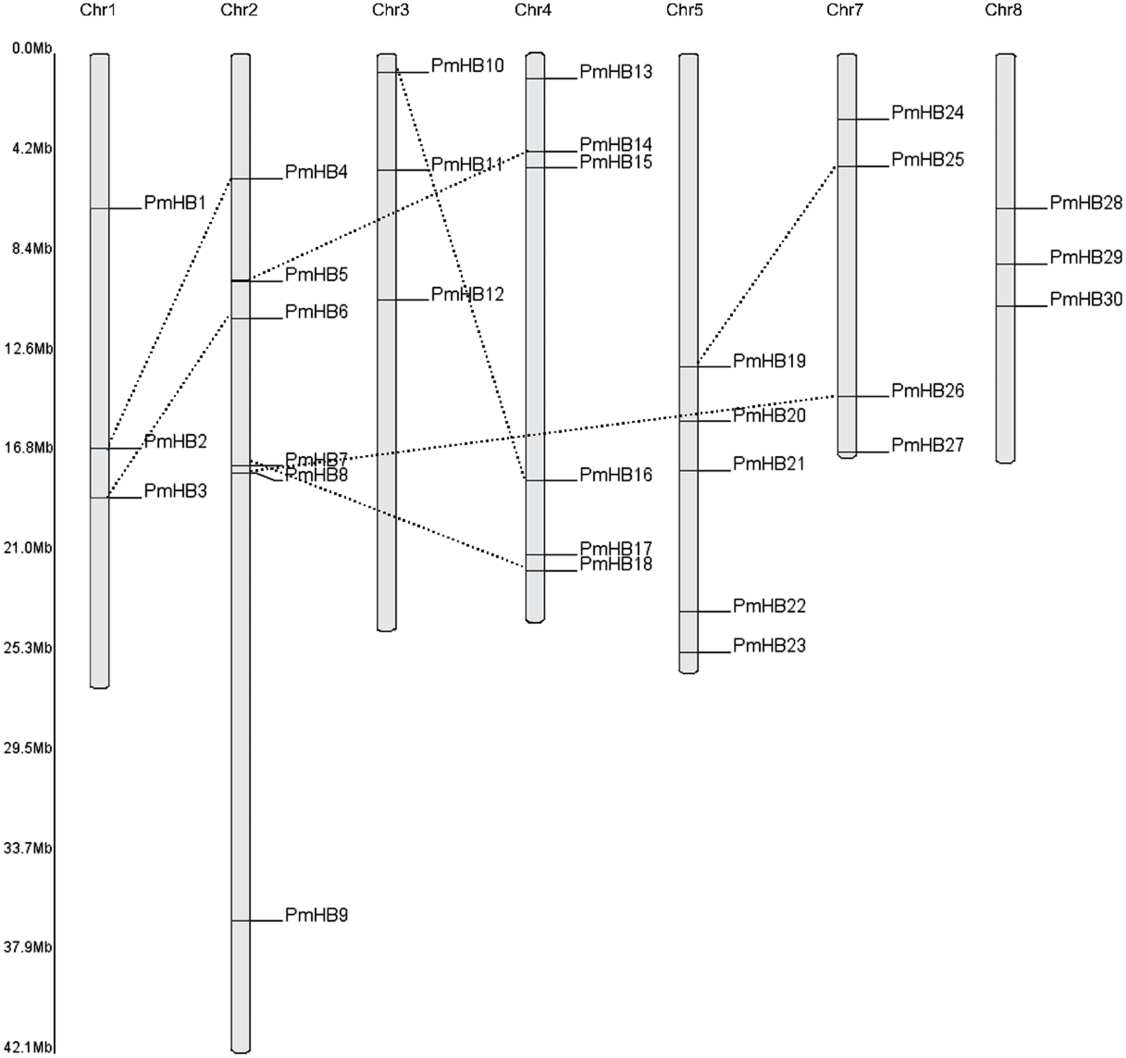
Chromosome localization of HD-Zip genes in *P. mume*. Thirty *PmHB* genes were located in seven chromosomes. The chromosome 6 has no *PmHB* genes. The dotted line indicates the duplicated gene pair.

Thirty of the *P. mume* HD-Zip genes were distributed among all the chromosomes, except for Chr6 (Fig. 2), and only two genes (*PmHB31* and *PmHB32*) remained on unmapped scaffolds. Chr2 and Chr4 contained the most HD-Zip genes, accounting for 18.75% (6) of the entire HD-Zip family; there were five genes on Chr5 and four on Chr7. In addition, Chr1, Chr3, and Chr8 each contained one gene (9.3%).

Because genome duplication is absent in *P. mume*, segmental duplication, tandem duplication, and transposons are the three mechanisms that resulted in gene expansion in *P. mume* (Clark & Donoghue, 2018; Del Pozo & Ramirez-Parra, 2015; Qiao et al., 2019). To identify potential segmental duplications in the HD-Zip family, pairs of *P. mume* and *P. persica* genes were obtained from the Plant Genome Duplication Database. The distribution of *PmHBs* relative to their corresponding duplicate genes is illustrated in Fig. 2 and Table 2. Among the 30 *PmHBs* mapped, seven *PmHB* gene pairs (43.8%) were found in segmental repeats (*PmHB2–PmHB4*, *PmHB8–PmHB26*, *PmHB7–PmHB18*, *PmHB3–PmHB6*, *PmHB5–PmHB14*, *PmHB10–PmHB16*, and *PmHB19–PmHB25*), representing all the HD-Zip subfamilies. No genes had been tandemly duplicated, suggesting that tandem duplications were not involved in the expansion of the HD-Zip gene family in *P. mume*.

**Table 2:** Estimated divergence period of HD-Zip gene pairs in *P. mume*.

**LOCUS 1**	**LOCUS 2**	**Ka**	**Ks**	**Ka/Ks**	**Select pressure**	**MYA**
*PmHB2*	*PmHB4*	0.3466	4.2421	0.081705	Purifying selection	141.40
*PmHB8*	*PmHB26*	0.3217	1.1706	0.274816	purifying selection	39.02
*PmHB7*	*PmHB18*	0.2416	1.2732	0.189758	purifying selection	42.44
*PmHB3*	*PmHB6*	0.3759	2.5532	0.147227	purifying selection	85.11
*PmHB5*	*PmHB14*	0.0941	1.1559	0.081408	purifying selection	38.53
*PmHB10*	*PmHB16*	0.1989	1.7398	0.114323	purifying selection	57.99
*PmHB19*	*PmHB25*	0.2835	1.2658	0.223969	purifying selection	42.19
*PmHB2*	*ppa008318m*	0.0089	0.0528	0.168561	purifying selection	1.76
*PmHB3*	*ppa008984m*	0.3583	2.0541	0.174432	purifying selection	68.47
*PmHB4*	*ppa009419m*	0.0083	0.0192	0.432292	purifying selection	0.64
*PmHB6*	*ppa009614m*	0.3838	2.4082	0.159372	purifying selection	80.27
*PmHB7*	*ppa020465m*	0.2535	1.2274	0.206534	purifying selection	40.91
*PmHB8*	*ppa018002m*	0.008	0.0405	0.197531	purifying selection	1.35
*PmHB9*	*ppa016510m*	0.0895	0.067	1.335821	positive selection	2.23
*PmHB12*	*ppa017711m*	0.0126	0.036	0.35	purifying selection	1.2
*PmHB17*	*ppa009747m*	0.1	0.1506	0.664011	purifying selection	5.02
*PmHB18*	*ppa009498m*	0.2416	1.37	0.17635	purifying selection	45.67
*PmHB19*	*ppa024161m*	0.0073	0.0497	0.146881	purifying selection	1.66
*PmHB21*	*ppa010647m*	0.007	0.0346	0.202312	purifying selection	1.15
*PmHB23*	*ppa011221m*	0.0236	0.0513	0.460039	purifying selection	1.71
*PmHB25*	*ppa008774m*	0.0099	0.0319	0.310345	purifying selection	1.06
*PmHB26*	*ppa008495m*	0.0039	0.0321	0.121495	purifying selection	1.07
*PmHB28*	*ppa008075m*	0.0038	0.0548	0.069343	purifying selection	1.83
*PmHB29*	*ppa015952m*	0.014	0.0503	0.27833	purifying selection	1.68
*PmHB30*	*ppa011343m*	0.01	0.0366	0.273224	purifying selection	1.22
*PmHB31*	*ppa011006m*	0.0873	0.1827	0.477833	purifying selection	6.09

Interspersed duplication, mainly attributable to transposons, was another mechanism that contributed to the expansion of the HD-Zip gene family. A total of 22 transposons or retrotransposons are found upstream and downstream of 15 *PmHB* genes. Details about the transposons and retrotransposons are shown in Table S2, implying that transposons may have played an important role in the expansion of HD-Zip genes in *P. mume*.

The ratio of nonsynonymous to synonymous mutation rates (Ka/Ks) was used to estimate the selection pressure that promoted the evolution of the gene family. The Ka, Ks, and Ka/Ks of 19 orthologous gene pairs between *P. mume* and *P. persica* and seven paralogous gene pairs in *P. mume* were calculated (Table 2). All the Ka/Ks of the paralogous gene pairs were <1, which indicates that the HD-Zip genes of *P. mume* have mainly experienced purifying selection pressure with limited functional divergence. In addition, most of the Ka/Ks ratios of the orthologous genes were <1, while the Ka/Ks value of *PmHB9/ppa016510 m* was >1, suggesting that HD-Zip genes evolved primarily under purifying selection that occurred after the divergence of *P. mume* and *P. persica*; adaptive evolution may have occurred in *PmHB9*.

### Conserved domains and gene structure analysis of *PmHBs*

**Figure 3:**
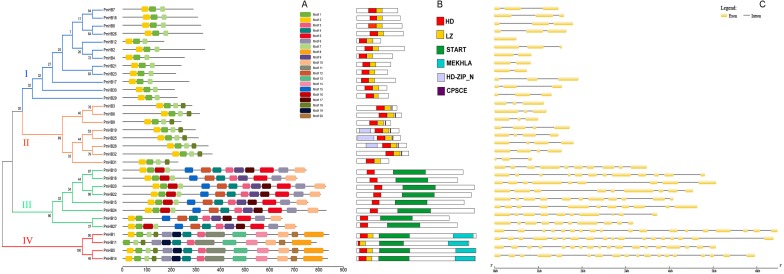
Conserved domains and gene structure analyses of HD-Zip genes in *P. mume*. (A) Conserved motif analysis of HD-Zip genes in *P. mume*; (B) conserved domains analysis of HD-Zip genes in *P. mume*; (C). intron/exon construction of HD-Zip genes in *P. mume*.

The conserved motifs of the HD-Zip protein sequences were analysed by MEME (Fig. 3A). Motif 1 and motif 2 were found to encode the homeobox domain. Motif 1 was found in all 32 genes. In addition, motif 7 was found to encode the LZ domain. Motifs 3, 4, 9, and 12 encode START domains and are present in subfamilies III and IV. Motif 8 was only found in subfamily III and encodes the MEKHLA domain. In addition, it was suggested that some unannotated subfamily-specific motifs might contribute to the diversification of the subfamily. A schematic representation of the HD-Zip family based on the HD domain, LZ domain, START domain and MEKHLA domain regions is shown in Fig. 3B.

The number of introns is similar in the different subfamilies (Fig. 3C). Most of the HD-Zip I genes have two introns, except *PmHB12*. In the HD-Zip II subfamily, *PmHB3*, *6*, *9*, and *31* have two introns, while *PmHB19*, *25*, *28*, and *32* have three introns. HD-Zip IV subfamily genes have 8–11 introns, whereas all the HD-Zip III genes have 17 introns, which was the maximum number among the HD-Zip family in *P. mume*. The results above divided the HD-Zip genes into four subfamilies, which were consistent with the result of the phylogenetic analysis.

### MicroRNA165/166 target-site prediction and cis-acting elements of the *PmHBs* promoters

**Figure 4:**
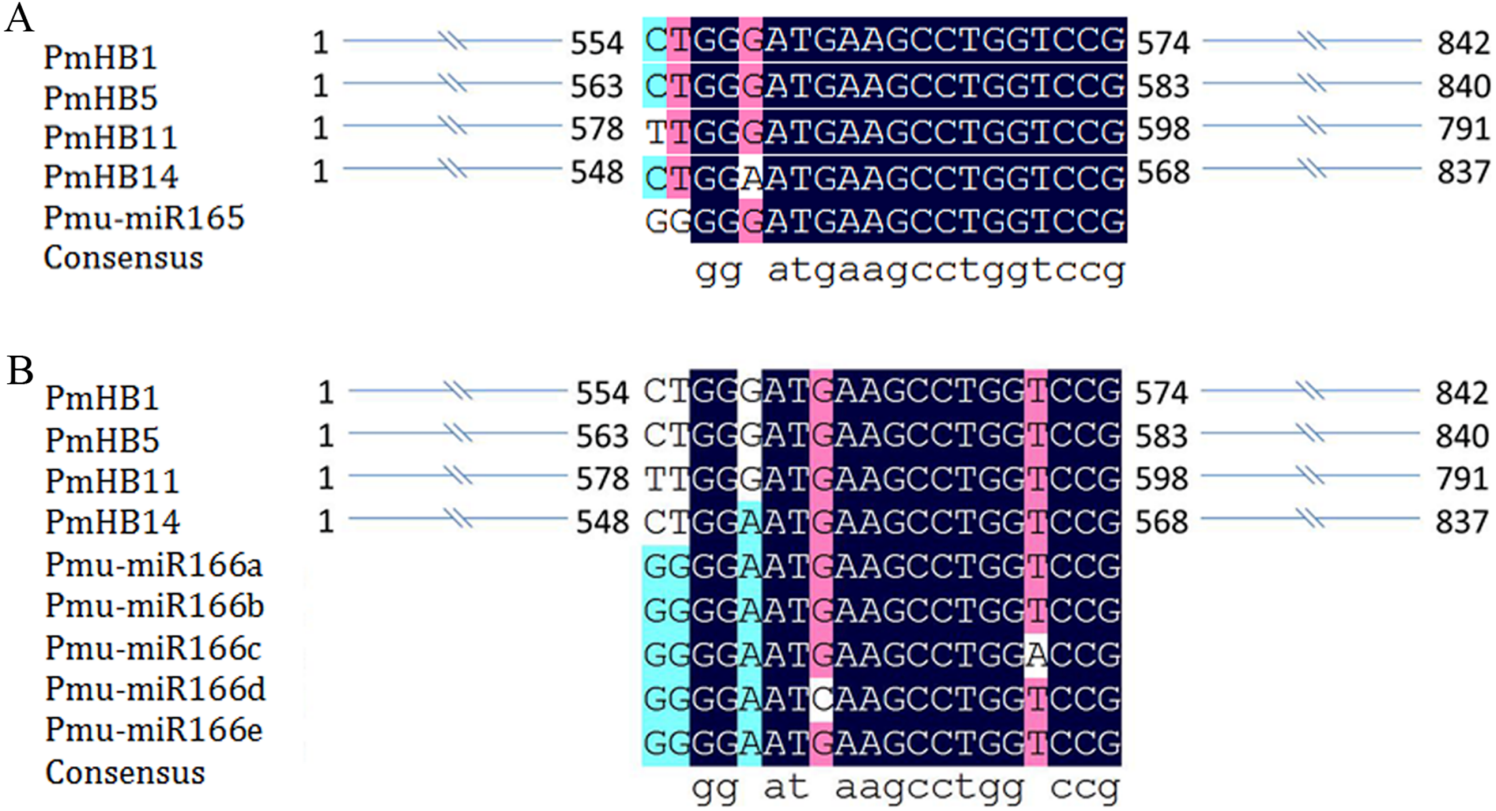
MiRNA target site in HD-ZIP III genes in *P. mume*. (A) miR165 target site in HD-ZIP III subfamily member; (B) miR166 target site in HD-ZIP III subfamily member and the secondary structures of miR166 a-e.

Previous studies revealed that the activity of HD-Zip III proteins was regulated by miR165/166 (Kidner & Martienssen, 2004; Mallory et al., 2014). Here, miR165 and miR166a–e target sites were found in four *P. mume* HD-Zip III subfamily genes (*PmHB1*, *PmHB5*, *PmHB11*, and *PmHB14*) (Fig. 4), suggesting that *P. mume* HD-Zip III subfamily genes may be regulated by miR165 and miR166.

**Figure 5:**
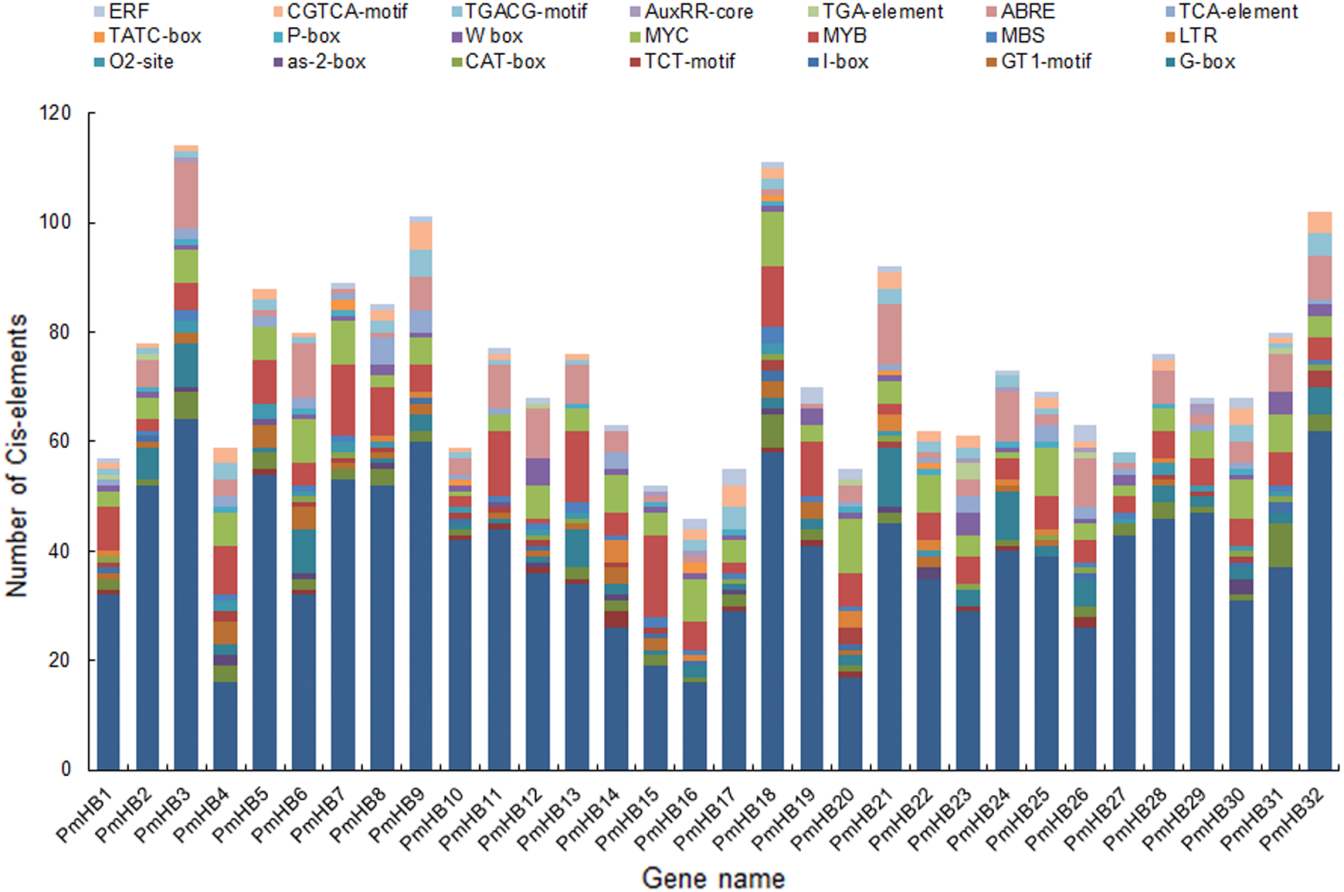
Types and number of cis-elements analysis involved in the stress response, hormone effects, and stem development. The *x*-axis represents 1.5 kb upstream promoter region of *PmHB* genes. The *y*-axis represents number of cis-promoters.

The cis-acting elements of promoters are essential for determining tissue-specific expression and are involved in the regulation of gene expression. A region of 1500 bp upstream of the start codon (ATG) of HD-Zip genes was used to analyse cis-acting regulatory elements. Many of the cis-acting elements identified in the *PmHB* gene promoters were related to the light response, including the AE-box, Box 4, the I-box, the GATA-motif, the G-box, the GT1-motif, and the TCT-motif (Fig. 5), suggesting that PmHBs may respond to light and participate in plant light morphogenesis. Because many cis-elements associated with hormone responses (the P-box, TATC-box, TCA-element, ABRE, TGA-element, AuxRR-core, TGACG-motif, CGTCA-motif, and ERF) were distributed in the promoter regions, the expression of *PmHB* genes may be induced by several plant hormones, such as salicylic acid, MeJA, abscisic acid, gibberellins, and auxin. In addition, the presence of shoot- and meristem-specific expression elements (as-2-box, CAT-box, and O2-site) in the promoters of several *PmHB* genes indicated that those genes might participate in regulating the development of the meristem and stem. HD-Zips are deemed to be crucial TFs involved in meristem and stem development.

### Expression analysis of *PmHBs* in different tissues of *P. mume*

**Figure 6:**
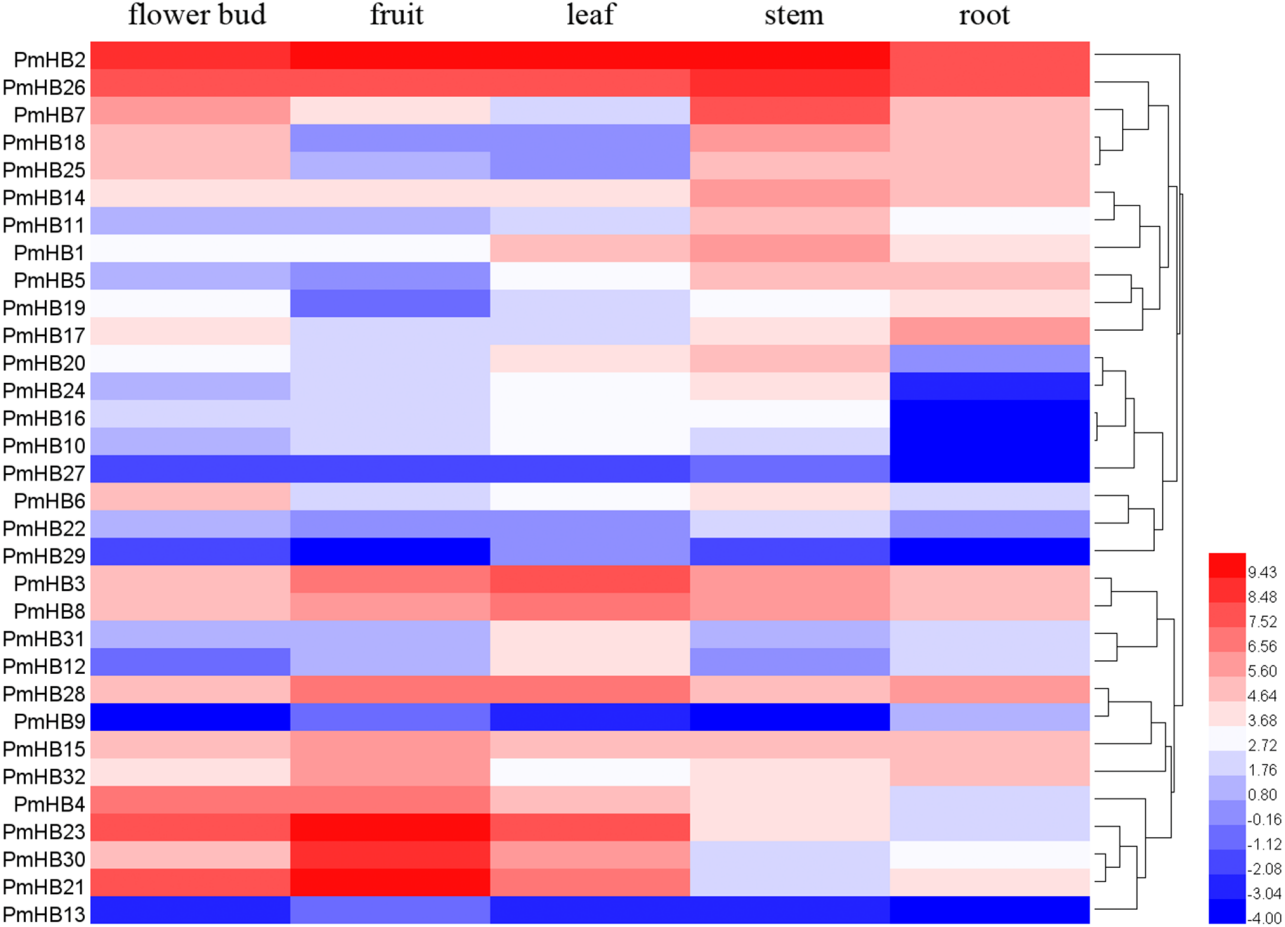
Hierachical clustering of expression profiles of *PmHBs* in leaf, flower bud, fruit, stem, and root. Color scale represents log2 rate expression values.

To explore the expression patterns of *PmHBs* in different organs and their biological functions during plant growth and development, the expression levels of *PmHBs* in five tissues (leaf, flower bud, fruit, root, and stem) were analysed by RNA-seq. Heatmaps showed that HD-Zip gene expression levels are diverse in the five tissues (Fig. 6). *PmHB29* transcripts were not present in fruits and roots, and *PmHB9* was not present in buds and stems; however, other HD-Zip genes could be detected in all five tissues. These results indicated that some HD-Zip genes have multiple functions in the development of different organs. *PmHB2* and *PmHB26* were highly expressed in all tissues, whereas *PmHB9*, *13*, and *27* were expressed at low levels in all tissues. Twelve genes (*PmHB1*, *5*, *7*, *11*, *14*, *16*, *18*, *20*, *22*, *25*, *26*, and *27*) were most highly expressed in the stem, which showed that these genes might have functions in regulating stem development. It is notable that *PmHB10*, *16*, *20*, and *24* were expressed in all aboveground organs but were expressed either at low levels or not at all in roots. In addition, some genes of the same subfamilies exhibited similar expression patterns. For instance, all HD-Zip III subfamily genes (*PmHB1*, *5*, *11*, and *14*) showed high RPKM values in the stem, and five HD-Zip IV subfamily genes (*PmHB16*, *17*, *20*, *22*, and *24*) showed high RPKM values in both leaf and stem tissues.

HD-Zip genes may also regulate flower bud dormancy. Screening for differentially expressed genes (DEGs) by RNA sequencing in flower buds at the endodormancy (ED) and natural flush (NF) stages was carried out in our previous study (Zhang et al., 2018). Eleven genes (*PmHB6*, *7*, *8*, *11*, *19*, *26*, *27*, *29*, *30*, and *31*) were more highly expressed at the ED stage, and four genes (*PmHB3*, *17*, *18*, and *23*) were upregulated at the NF stage (Table S3).

### Expression pattern analysis of *PmHBs* in stem development

To verify the functions of *PmHBs* in the development of leaf buds and stems, RNA-seq on leaf buds and stem tips was conducted in *P. mume*. HD-Zip family genes that were differentially expressed the between leaf bud and stem tip are shown in Table S4. Seven genes (*PmHB10*, *16*, *20*, *22*, *24*, *27*, and *29*) were upregulated in leaf buds, while another six genes (*PmHB3*, *13*, *17*, *18*, *21*, and *30*) were upregulated in stem tips. The results above suggest that HD-Zip genes play putative roles in the development of leaf buds and stems.

**Figure 7:**
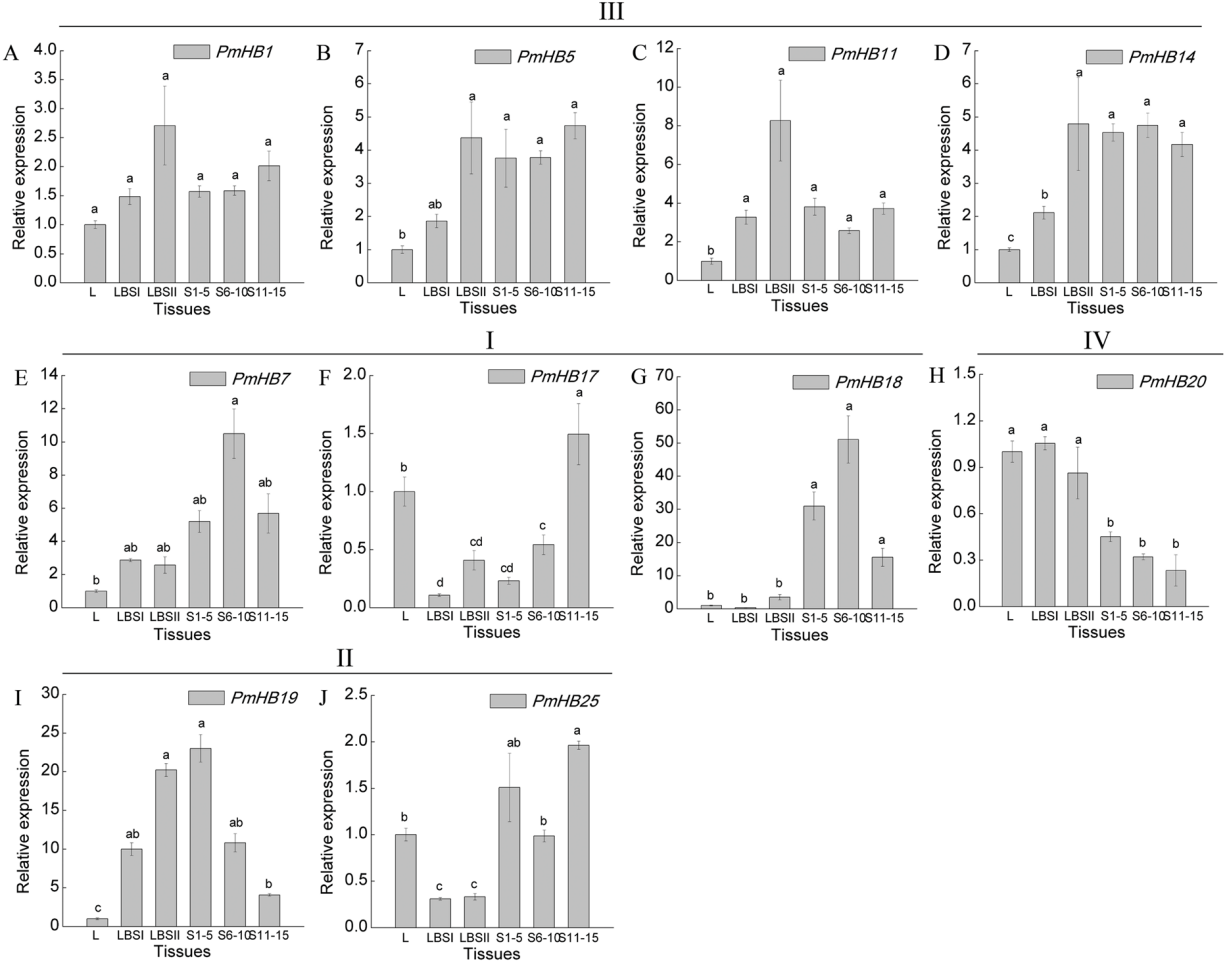
Expression pattern analysis of HD-Zip genes in *P. mume* at different development stages. Ten HD-Zip genes were selected to analysis the expression patterns in leaf, leaf buds, and stems at different development stages (A-J). L, leaf; LBSI (leaf buds in stage I), leaf bud in sprouted phase; LBSII (Leaf bud in stage II), leaf buds before leaf unfolding; S1-5, stems from 1 to 5 internodes; S6-10, stems from 6 to 10 internodes; S11-15, stems from 11 to 15 internodes. *PmPP2A and Pmactin* were used as reference genes. Vertical lines represent S.E. (*n* = 3). Different lower case letters indicate statistically significant differences at the *P* < 0.01 level.

Expression levels of 10 selected genes in different organs (leaf, leaf bud, and differentiating stem) were determined by qRT-PCR (Fig. 7). The results showed that genes in the HD-Zip III subfamily had similar expression patterns, which were highly expressed in both LBSII and stems, and these patterns were not significantly different at different stages of stem development; *PmHB7* and *PmHB18*, two HD-Zip I genes, were predominantly expressed in S6–10 in stem, while another HD-Zip I gene, *PmHB17*, exhibited high levels of expression in leaf and in S11–15. *PmHB20* of the HD-Zip IV subfamily had higher expression levels in leaves, leaf buds, and large leaf buds and lower expression levels in different stages of stem development. *PmHB19* is a HD-Zip II gene with high expression levels in both large leaf buds and stem tips (S1–5). Another HD-Zip II gene, *PmHB25*, was expressed maximally in S11–15, while it exhibited its lowest expression levels in LBSI and LBSII (Fig. 7).

**Figure 8:**
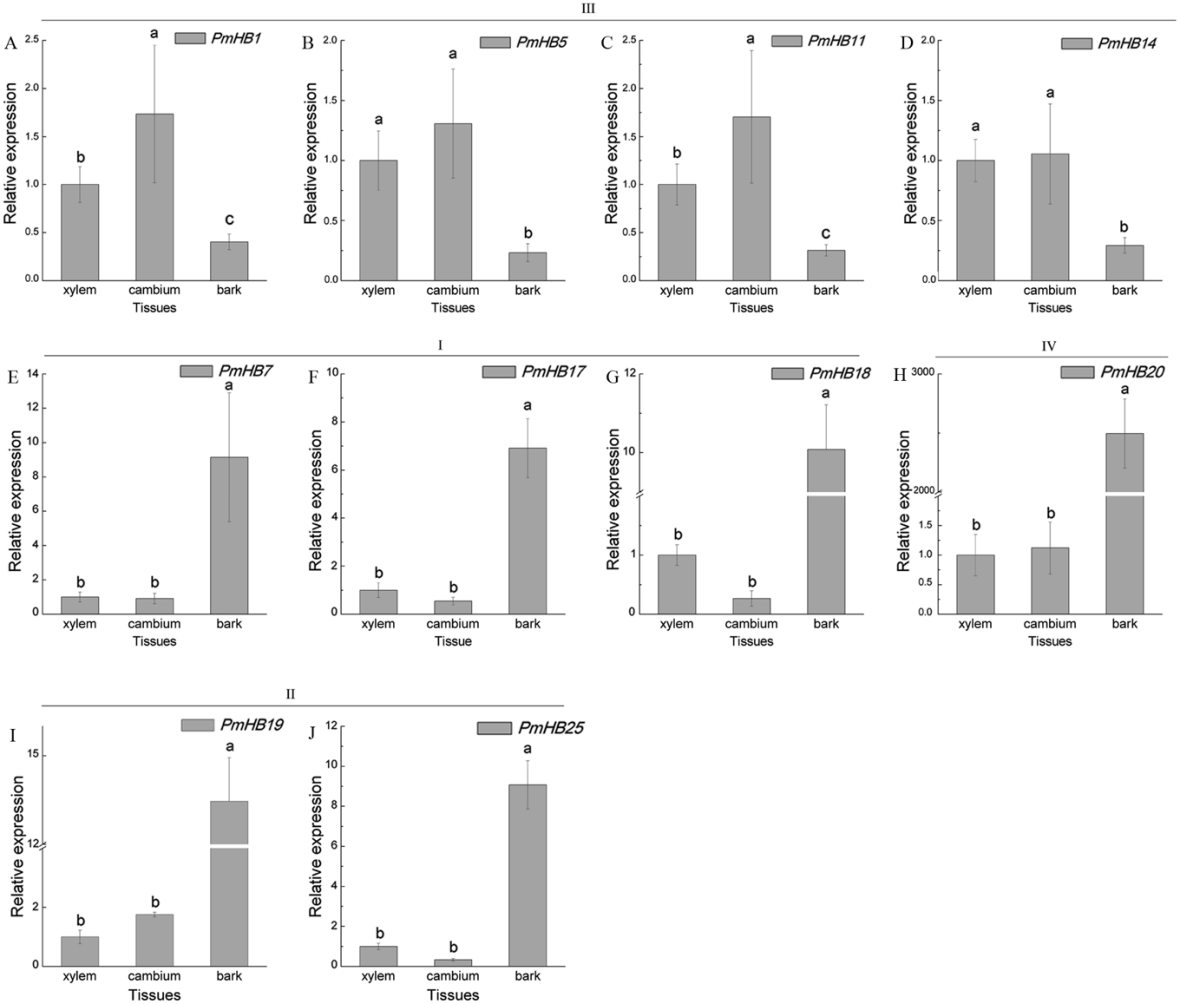
Expression pattern analysis of HD-Zip genes in *P. mume* in different tissues of young stems. Ten HD-Zip genes were selected to analysis the expression patterns in different tissues of young stems (A-J). *PmPP2A and Pmactin* were used as reference genes. Vertical lines represent S.E. (*n* = 3). Different lower case letters indicate statistically significant differences at the *P* < 0.01 level.

Expression in different tissues of the stem (xylem, cambium, and bark) were determined by qRT-PCR (Fig. 8). HD-Zip III subfamily genes (*PmHB1*, *5*, *11*, and *14*) may participate in xylem differentiation because they are expressed predominantly in the xylem. However, *PmHB7*, *17*, *18*, *19*, *20*, and *25* were mainly expressed in bark, suggesting that they might be involved in the development of phloem, endodermis, or epidermis (Fig. 8).

### Interaction network between ten *P. mume* and *A. thaliana* HD-Zip proteins

The GO annotation of HD-Zip genes in *P. mume* were conducted by Blast2GO software basing on the orthologues in *Arabidopsis* (Fig. S2). *PmHBs* were divided into three GO categories: biological processes, cell components and molecular functions. In the biological process category, biological regulation was the most highly represented groups. The cell, cell part, and organelle classes were abundant in cellular component category. Within the molecular function category, genes that corresponded to binding were the most abundant.

**Figure 9:**
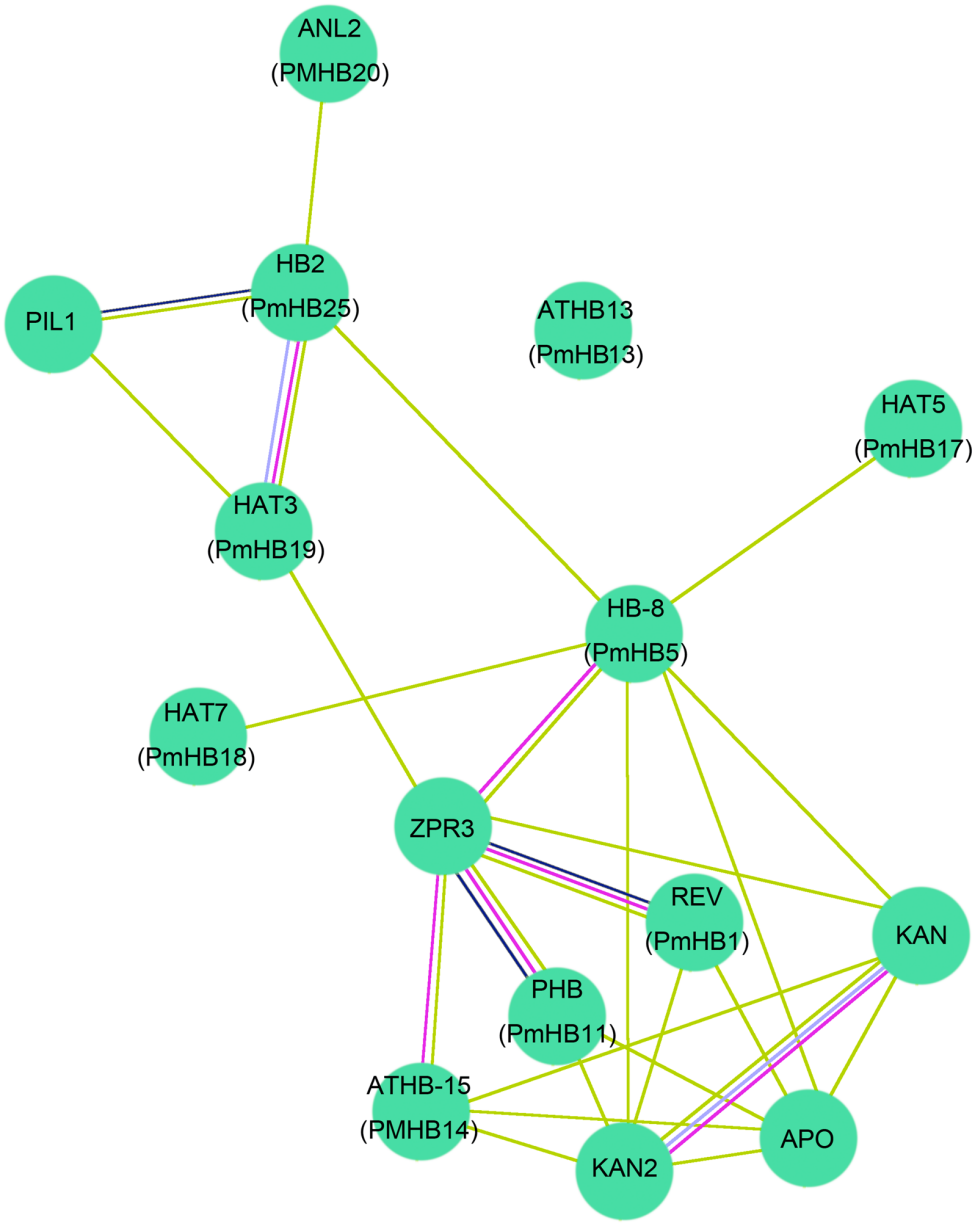
Interaction networks of ten HD-Zip proteins in *P. mume* according to the orthologs in *Arabidopsis*. The nodes with different colors represent different proteins and the lines with different color represent different types of evidence for the interaction. Light blue lines show known interactions from curated databases, rose red lines show known interactions experimentally determined, blue lines show predicted interactions of gene co-occurrence, black lines show interactions of co-expression, and yellow lines show interactions of text mining.

To further predict *P. mume* HD-Zip proteins functions during plant stem development, the protein–protein interactions between ten HD-Zip proteins of *P. mume* and *A. thaliana* were analysed using STRING software (http://string-db.org) (Fig. 9). The homologous genes were matched with the highest bit score, the results were corresponding to those of Blast2GO and the lines of different colours represent different types of evidence for the proteins interaction. Ten HD-Zip proteins were found to interact in a network, suggesting that they might have complex connections in the co-regulation of stem development. Previous studies showed that HD-Zip III activity can promote the expression of ZPR proteins in *A. thaliana* (Husbands et al., 2016; Wenkel et al., 2007). Specifically, REV and PHB can interact with ZPR3 to participate in the regulation of leaf polarity and vascular development (Kim et al., 2008; Wenkel et al., 2007). Moreover, interactions between the HD-Zip III and KANDAI proteins to regulate vascular development have been reported in *A. thaliana* (Emery et al., 2003a; Emery et al., 2003b). The HD-Zip III subfamily genes *PmHB1*, *5*, *11*, and *14* had highly similar amino sequences to those of *REV*, *HB-8*, *PHB*, and *ATHB-15*, respectively, and might regulate leaf and vascular development. In addition, HB-2 was induced by the PIFs and participated in the shade avoidance response (Kunihiro et al., 2011). *PmHB25*, which is closely related to *HB-2*, may play an important role in regulating light-mediated stem elongation.

## Discussion

HD-Zip, containing a homeodomain (HD) domain and an adjacent leucine zipper (LZ), is a major group of transcriptional factors present in higher plants that has a significant impact on the light reduction, abiotic stress, and organic development processes of plants. Most studies on HD-Zip proteins have focused on herbaceous plants, while the functions of only a few HD-Zip proteins have been verified in woody plants, such as poplar and peach (Hu et al., 2012; Zhang et al., 2014a; Zhang et al., 2014b). However, a great deal is unknown about these genes in *P. mume*. A total of 32 genes were identified, which is 0.7-fold more than the number found in *Arabidopsis* (Emery et al., 2003b; Henriksson, 2005; Nakamura et al., 2006; Ciarbelli et al., 2008), 0.5-fold more compared to *Populus* (Hu et al., 2012), and similar to the number found in *P. persica* (Zhang et al., 2014a; Zhang et al., 2014b). The number of HD-Zip genes varies widely among these species. Nevertheless, the *P. mume* genome (280 Mb) is approximately two-fold larger than that of *Arabidopsis* (125 Mb) (Zhang et al., 2012; Initiative, 2000). To explore the phylogenetic relationship between the HD-Zip proteins of *P. mume* and *Arabidopsis*, *PmHB* orthologues in *Arabidopsis* with the highest levels of homology were extracted (Table 1), showing that a single HD-Zip gene in *Arabidopsis* had multiple orthologues in *P. mume*. For instance, *ATHB-6*, *HDG11*, *ANL2*, and *HOX11* had two orthologues each in *P. mume*, whereas *HAT22* and *HAT3* had three orthologues each in *P. mume*. These results suggested that, such as peach, some HD-Zip genes may have been lost in the *P. mume* lineage during evolution (Zhang et al., 2014a; Zhang et al., 2014b). The number of HD-Zip genes in *P. mume* was similar to that in peach, which might be because of their closer genetic relationship.

The 32 putative genes were classified into four subfamilies according to the results of phylogenetic analysis, and the length and intron number of the genes in different subfamilies were conserved (Figs. 1 and 3). For example, the lengths of HD-Zip I and II genes were shorter than those of HD-Zip III and IV. HD-Zip III genes have 17 introns. In addition, proteins in the same subfamily showed similar structures. In general, most of the motifs and domains in the same subfamily were similar, which may be consistent with the phylogenetic analysis. However, some motifs were specific to subfamilies. For example, motif 18 was only detected in the HD-Zip II subfamily; motifs 3 and 4 were found in the HD-Zip III and IV subfamilies, and motifs 5, 6, 9, 10, 12, 15, and 17 were only found in the HD-Zip IV subfamily, which might be associated with the functional diversity of the HD-Zip family. These findings clearly support the results of phylogenetic analysis. Tandem duplication, segmental duplication, and transposons have resulted in the genome evolution. It was revealed that segmental duplication and transposons may have contributed to the expansion of the HD-Zip gene family in *P. mume*, accounting for 92% of duplications (Fig. 2 and Table 2).

Proteins can interact with other proteins to co-regulate biological processes. A network of ten *P. mume* and *Arabidopsis* HD-Zip TF pairs was established. In response to shade, plants can react by altering elongation during growth and development. It has been found that HD-Zip genes positively regulate the plant response to shade. In *Arabidopsis*, the HD-Zip II gene *HB-2* can be activated by PIF4 and PIF5, two phytochrome-interacting transcriptional factors that participate in the regulation of hypocotyl elongation under short-day conditions (Kunihiro et al., 2011). As the orthologues of *HB-2* and *HAT3*, *PmHB25* and *PmHB19* may also play roles in shade avoidance. Moreover, *Arabidopsis hat3/athb4/athb2* loss-of-function triple mutants showed developmental defects in white light, such as adaxialized leaves and reduced shoot apical meristem activity (Turchi et al., 2013). Previous research showed that the expression of several HD-Zip II transcriptional factors, including *HAT3* and *HB-2/HAT4*, was regulated by the HD-Zip III gene *REVOLUTA* (*REV*) (Bou-Torrent et al., 2012). Mutation of *REV*, *PHABULOSA* (*PHB*), and *PHAVOLUTA* (*PHV*) enhanced the bilateral symmetry and SAM defects in *hat3/athb4* mutants. These results indicated that HD-Zip II and HD-Zip III genes cooperate in establishing bilateral symmetry and patterning in the embryo, as well as in controlling SAM activity in plants. Six genes in both the HD-Zip II and III subfamilies (*PmHB1*, *5*, *11*, *14*, *19*, and *25*) had higher expression levels in leaf buds before leaf unfolding (LBSII) or in stem tips (S1–5), suggesting that *PmHB1*, *5*, *11*, *14*, *19*, and *25* might participate in the formation and maintenance of SAM polarity in *P. mume*.

HD-Zip III transcriptional factors also play roles in controlling primary and secondary vascular cell differentiation and SAM (shoot apical meristem) maintenance (Baima, 2001; Kim et al., 2005), which is essential for the formation of plant architecture. Overexpression of *ATHB-8* promotes vascular cell differentiation and xylem tissue production in inflorescence stems of Arabidopsis (Baima, 2001). HD-Zip III TFs are key factors that regulate the secondary growth of vascular bundles in *Populus*. *PtrHB7*, an *ATHB-8* homologue, regulates the balance of vascular patterning by inhibiting the differentiation of secondary xylem while promoting the differentiation of secondary phloem (Zhu et al., 2013). *REV*, which has overlapping functions with *PHV* and *PHB*, regulates meristem initiation in lateral organs in opposition to *KANADI* (Ilegems et al., 2010). Overexpression of *PRE*, the *Populus* homologue of the *Arabidopsis REV* gene, in *Populus* causes the patterning of secondary vascular tissues to be altered (Robischon et al., 2011). In addition, genes of the four HD-Zip III subfamilies of *P. mume* were expressed preferentially in xylem and cambium; thus, we speculated that four genes from the HD-Zip III subfamily were closely associated with stem development in *P. mume*. The expression of HD-Zip III genes was controlled by multiple molecular mechanisms. First, the small ZIP protein ZPR3 reduced HD-Zip III protein activity by direct interaction to form nonfunctional heterodimers in the SAM of *Arabidopsis* (Kim et al., 2008). Moreover, overexpression of *KANADI* negatively affected the expression of HD-Zip III genes during embryogenesis, whereas HD-Zip III genes and *KANADI* co-regulated auxin flow by controlling the distribution of PIN-FORMED1 (PIN1) (Izhaki & Bowman, 2007). In addition, protein-interaction networks showed that *REV*, *HB-8*, *PHB*, and *ATHB-15*, the orthologues of *PmHB1*, *PmHB5*, *PmHB11*, and *PmHB14*, respectively, interacted with ZPR3, and ATHB-15 interacted with KANADI (Fig. 9). Finally, MiR165/166 negatively regulated the activity of HD-Zip III genes in both *Arabidopsis* (Kim et al., 2005; Mallory et al., 2014) and *Populus* (Ko, Prassinos & Han, 2006). Because the target sites were present in the sequences of HD-Zip III genes, we speculated that HD-Zip III genes might also be regulated by MiR165/166. However, the molecular mechanisms of the HD-Zip III gene regulation of vascular development and interactions between proteins need further investigation.

HD-Zip IV proteins have been reported to regulate the differentiation and maintenance of outer cell layers (Nakamura et al., 2006; Yan et al., 2018). PDF2 and ATML1 regulate shoot epidermal cell differentiation and embryo development in *Arabidopsis* (Ogawa et al., 2015). In *P. mume*, the expression level of *PmHB20* in bark was more than 2,000 times that in cambium and xylem (Fig. 8), which is similar to *Arabidopsis* HD-Zip IV genes that are preferentially or specifically expressed in plant epidermal or sub-epidermal cells (Rombolácaldentey et al., 2014).

## Conclusions

Thirty-two HD-Zip genes were identified in *P. mume*. Phylogenetic analysis showed that there were four subfamilies in the HD-Zip family: subfamilies I, II, III, and IV. Gene structure and motif analysis suggested that genes in the same subfamily were conserved. Furthermore, expression profile analysis showed that HD-Zip genes may be involved in stem development and flower bud dormancy. Otherwise, all HD-Zip III genes were highly expressed in cambium and xylem, suggesting that HD-Zip III genes may be indispensable in vascular differentiation. The prediction of microRNA target sites indicated that HD-Zip III subfamily genes may be regulated by miR165/166. Promoter analysis showed that HD-Zip III genes may be involved in responses to light, hormones, and abiotic stressors, as well as plant development. Our study identified the members of the HD-Zip family in *P. mume* and laid the foundation for molecular breeding programmes for woody ornamental plants.

##  Supplemental Information

10.7717/peerj.7499/supp-1 Table S1 Sequence of forward and reverse primer pairs for real-time quantification PCR

10.7717/peerj.7499/supp-2 Table S2 Transposons and retrotransposons in genomic sequences of 10-kb upstream and downstream of each HD-ZIP gene

10.7717/peerj.7499/supp-3 Table S3 Differentially expressed genes (DEGs) in flower buds with endodormancy stage (ED) and natural flush stage (NF) in *P. mume*

10.7717/peerj.7499/supp-4 Table S4 Differentially expressed genes (DEGs) in leaf bud and stem tip *of P. mume*

10.7717/peerj.7499/supp-5 Figure S1 Samples of leaf buds and young stems in different stages L, leaf; LBSI (leaf buds in stage I), leaf bud in sprouted phase; LBSII (Leaf bud in stage II), leaf buds before leaf unfolding; S1-5, stems from 1 to 5 internodes; S6-10, stems from 6 to 10 internodes; S11-15, stems from 11 to 15 internodes

10.7717/peerj.7499/supp-6 Figure S2 GO annotation of *HD-Zip* genes in *P. mume*

10.7717/peerj.7499/supp-7 Data S1 Protein sequences of HD-Zip family in *A. thaliana*, *O. sativa* and* P. mume*

10.7717/peerj.7499/supp-8 Data S2 The detailed information of all orthologues in *Arabidopsis*

## Additional Information and Declarations

### Data Availability

The following information was supplied regarding data availability: The raw data is available at NCBI via accession number GCA_000346735.1 (https://www.ncbi.nlm.nih.gov/genome/13911?genome_assembly_id=44389).
