# Supplementary material for: Genome-wide identification, characterization and expression analysis of the HD-Zip gene family in the stem development of the woody plant Prunus mume
Source: PeerJ. 2019 Aug 8;7:e7499. doi: 10.7717/peerj.7499 (PMC6689393; doi:10.7717/peerj.7499)
Supplement: Table S1 [file peerj-07-7499-s001.doc]

**Table S1** Sequence of forward and reverse primer pairs for real-time quantification PCR

| Gene | Primers sequences |
| --- | --- |
| *PmHB1-F* | ATCTCTTTCTGGTTCTGGCG |
| *PmHB1-R* | ACTCCATGCCTCAAGATTCAG |
| *PmHB5-F* | GAGTGGATGAGAATTCGGTCG |
| *PmHB5-R* | AGAGGCAAGATCAAGTGTACG |
| *PmHB11-F* | TGGTTCCCAGTATAACGCATC |
| *PmHB11-R* | ATCAACCCCATAATCAGCCC |
| *PmHB14-F* | TTATTTCCGTCAGCATACCCAG |
| *PmHB14-R* | AGCAGTTCCAGTAGCCTTTG |
| *PmHB7-F* | TGAAAAGTAGAGAACCAGCGG |
| *PmHB7-R* | GGATGAGTAGATTGAGGGCTG |
| *PmHB17-F* | CAAAGTTGAGGACCGTCTGAG |
| *PmHB17-R* | AGAAAATTGTAGGAGTCGCCG |
| *PmHB18-F* | CCTCCACCTTGCTAATTCCTC |
| *PmHB18-R* | CCTCCATGCAGTCCTACTTC |
| *PmHB19-F* | AGAGGTGGATTGTGAGTTGC |
| *PmHB19-R* | GAAGATGACAAGGATGAGACGG |
| *PmHB20-F* | GTCCATGTTCAACTCTCCAGG |
| *PmHB20-R* | ATTCTCAGCCATTCTCGTCAC |
| *PmHB25-F* | TTTTCCTCAGACCCACAACC |
| *PmHB25-R* | AAGCAGAAGTAGGGACCAAAG |
| *Actin-F* | CCCTAAGGCTAACAGAGGAAACA |
| *Actin-R* | CATACATGGCAGGCACATTGAAG |
| *PP2A-F* | AGGGTTCGGCTCGCAATAATAGA |
| *PP2A-R* | AGCAGCAGCATCACGAATTGAGTAG |
