# Supplementary material for: Genome-wide identification, characterization and expression analysis of the HD-Zip gene family in the stem development of the woody plant Prunus mume
Source: PeerJ. 2019 Aug 8;7:e7499. doi: 10.7717/peerj.7499 (PMC6689393; doi:10.7717/peerj.7499)
Supplement: Table S2 [file peerj-07-7499-s002.doc]

**Table S2** Transposons and retrotransposons in genomic sequences of 10-kb upstream and downstream of each HD-ZIP gene

|  | Gene name | class/family |
| --- | --- | --- |
| upstream | PmHB5 | LTR/Cassandra |
|  | PmHB8 | LTR/Copia |
|  | PmHB8 | LTR/Copia |
|  | PmHB8 | LTR/Copia |
|  | PmHB8 | LTR/Copia |
|  | PmHB13 | LTR/Cassandra |
|  | PmHB13 | LTR/Copia |
|  | PmHB15 | DNA/PIF-Harbinger |
|  | PmHB16 | LTR/Copia |
|  | PmHB18 | LTR/Copia |
|  | PmHB21 | DNA/MULE-MuDR |
|  | PmHB22 | DNA/MULE-MuDR |
|  | PmHB25 | LTR/Gypsy |
| downstream | PmHB2 | LTR/Cassandra |
|  | PmHB2 | LTR/Gypsy |
|  | PmHB3 | LTR/Copia |
|  | PmHB10 | LTR/Copia |
|  | PmHB23 | LTR/Gypsy |
|  | PmHB24 | LTR/Copia |
|  | PmHB30 | LTR/Gypsy |
|  | PmHB30 | LTR/Copia |
|  | PmHB30 | LTR/Copia |
