# Supplementary material for: Genome-wide identification, characterization and expression analysis of the HD-Zip gene family in the stem development of the woody plant Prunus mume
Source: PeerJ. 2019 Aug 8;7:e7499. doi: 10.7717/peerj.7499 (PMC6689393; doi:10.7717/peerj.7499)
Supplement: Table S3 [file peerj-07-7499-s003.docx]

**Table S3** Differentially expressed genes (DEGs) in flower buds with endodormancy stage (ED)and natural flush stage (NF) in *P. mume*

| Gene name | ED _FPKM | NF _FPKM |
| --- | --- | --- |
| PmHB3 | 10.37203 | 29.55664 |
| PmHB17 | 4.261885 | 12.32421 |
| PmHB18 | 8.91443 | 41.45136 |
| PmHB23 | 6.164975 | 27.62633 |
| PmHB6 | 11.68243 | 4.183708 |
| PmHB7 | 135.0367 | 57.77235 |
| PmHB8 | 39.31467 | 17.79453 |
| PmHB11 | 71.27054 | 23.09897 |
| PmHB19 | 30.37679 | 7.316407 |
| PmHB26 | 315.7605 | 130.3903 |
| PmHB27 | 14.37863 | 3.480675 |
| PmHB29 | 4.463077 | 0.757585 |
| PmHB30 | 13.77594 | 3.712228 |
| PmHB31 | 1.713618 | 0.445394 |

ED_RPKM and NF_RPKM means the reads per kilobase per million (RPKM) values of individual gene in endodormancy stage and natural flush stage, respectively.
