# Supplementary material for: Genome-wide identification, characterization and expression analysis of the HD-Zip gene family in the stem development of the woody plant Prunus mume
Source: PeerJ. 2019 Aug 8;7:e7499. doi: 10.7717/peerj.7499 (PMC6689393; doi:10.7717/peerj.7499)
Supplement: Table S4 [file peerj-07-7499-s004.docx]

**Table S4** Differentially expressed genes (DEGs) in leaf bud and stem tip *of P. mume*

| **Gene** | **leaf bud_RPKM** | **stem tip_RPKM** |
| --- | --- | --- |
| *PmHB3* | 9.24892 | 29.09833 |
| *PmHB10* | 13.33881 | 2.523191 |
| *PmHB13* | 0.098307 | 0.775947 |
| *PmHB16* | 27.75317 | 3.500433 |
| *PmHB17* | 4.093003 | 18.01549 |
| *PmHB18* | 14.4258 | 30.44297 |
| *PmHB20* | 74.04256 | 32.29551 |
| *PmHB21* | 2.86101 | 5.932537 |
| *PmHB22* | 4.347459 | 0.957368 |
| *PmHB24* | 22.29131 | 3.390858 |
| *PmHB27* | 7.895008 | 0.896778 |
| *PmHB29* | 12.30841 | 4.480275 |
| *PmHB30* | 3.902162 | 12.69439 |

Leaf bud_RPKM and stem tip_RPKM mean the reads per kilobase per million (RPKM) values of individual gene in leaf bud and stem tip *of P. mume*, respectively.
