# Supplementary figures and images for: Genome-wide identification, characterization and expression analysis of the HD-Zip gene family in the stem development of the woody plant Prunus mume
Source: PeerJ. 2019 Aug 8;7:e7499. doi: 10.7717/peerj.7499 (PMC6689393; doi:10.7717/peerj.7499)

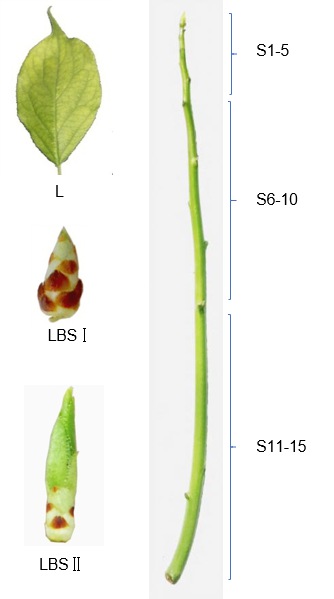

Supplement: Figure S1 — L, leaf; LBSI (leaf buds in stage I), leaf bud in sprouted phase; LBSII (Leaf bud in stage II), leaf buds before leaf unfolding; S1-5, stems from 1 to 5 internodes; S6-10, stems from 6 to 10 internodes; S11-15, stems from 11 to 15 internodes [file peerj-07-7499-s005.jpg]

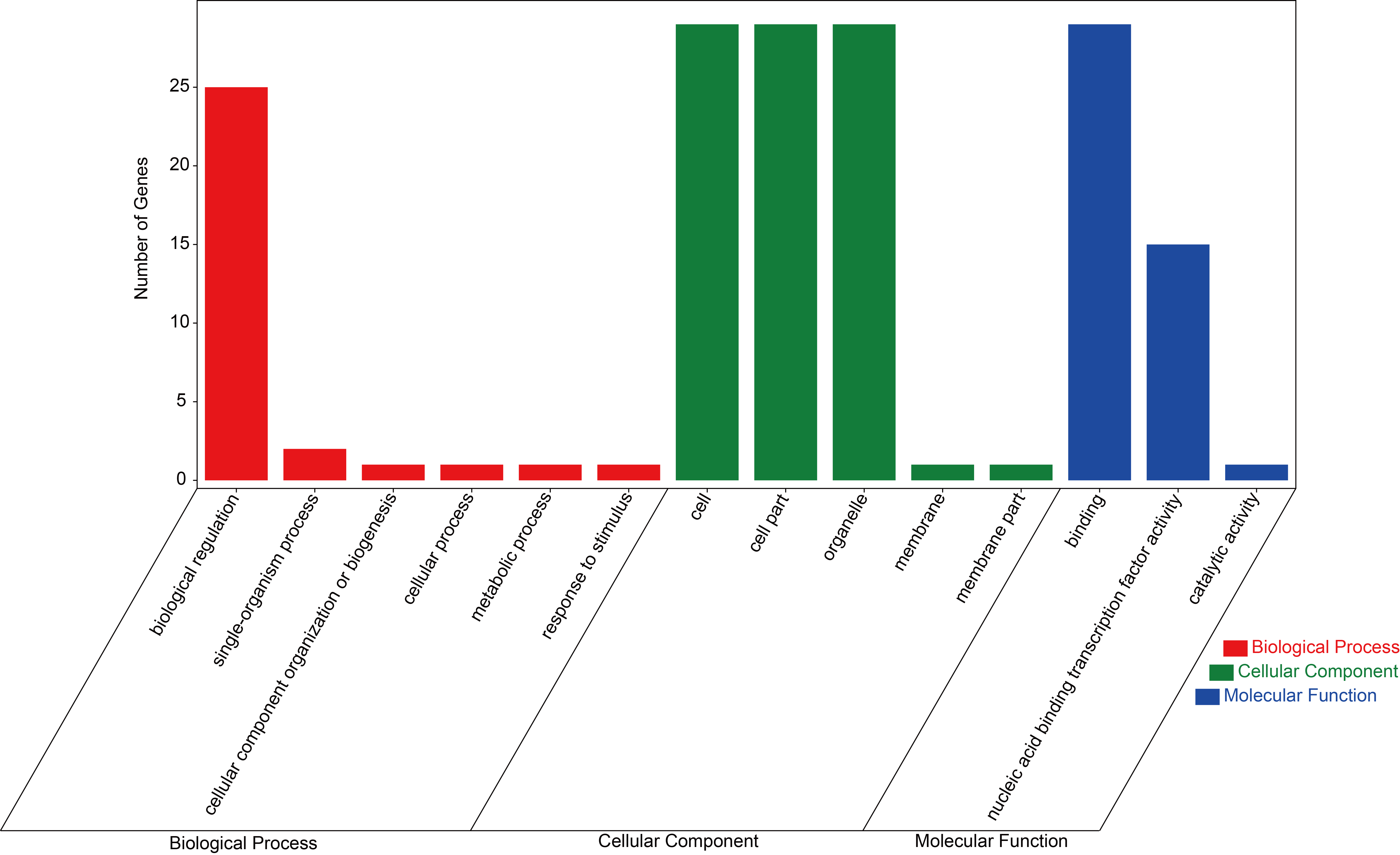

Supplement: Figure S2 [file peerj-07-7499-s006.png]
